# Absence of Middle East respiratory syndrome coronavirus in Bactrian camels in the West Inner Mongolia Autonomous Region of China: surveillance study results from July 2015

**DOI:** 10.1038/emi.2015.73

**Published:** 2015-12-02

**Authors:** Renqiang Liu, Zhiyuan Wen, Jinling Wang, Jinying Ge, Hualan Chen, Zhigao Bu

**Affiliations:** 1 State Key Laboratory of Veterinary Biotechnology, Harbin Veterinary Research Institute, Chinese Academy of Agricultural Sciences, Harbin 150001, Heilongjiang Province, China; 2 College of Veterinary Medicine, Inner Mongolia Agricultural University, Hohhot 010018, Inner Mongolia Autonomous Region, China

## 

**Dear Editor,**

Middle East respiratory syndrome coronavirus (MERS-CoV) was first identified among humans in Saudi Arabia in 2012.^1^ As of 2 September 2015, a total of 1545 MERS cases and 588 deaths had been confirmed.^2^ Most cases have occurred in Saudi Arabia and the United Arab Emirates. Of note, a major MERS outbreak in South Korea caused 186 cases and 36 deaths in 2015.^3^ China also had one confirmed case imported from South Korea. Studies have confirmed the presence of MERS-CoV in dromedaries in the Arabian Peninsula and North Africa.^4,5^ Dromedaries are thought to be the main reservoir of MERS-CoV. Although transmission of MERS-CoV from camels to humans has not been reported to date, it has been postulated that primary human infection could result from close contact with camels, which shed virus.^6^

There are two kinds of camels: one-hump dromedaries (*Camelus dromedarius*) and two-hump Bactrian camels (*Camelus bactrianus*). Dromedaries are mainly found in the Arabian Peninsula, the Middle East, and parts of Africa, whereas Bactrian camels are mainly located in central and northeast Asia, northern China, and Mongolia. A surveillance study conducted by Chan *et al.* indicated that MERS-CoV was not present in Bactrian camels in Mongolia.^7^ China has a very long history of camel raising. To date, there are an estimated 300 000 Bactrian camels in China, over 150 000 of which are distributed across the desert steppe of the West Inner Mongolia Autonomous Region (IMAR). In the West IMAR, Alxa, Bayan Nur, and Ordos have approximate populations of 100 000, 18 000, and 5000 camels, respectively; these three areas thus hold over 40% of the Bactrian camels in China. Compared to Mongolia, West IMAR has a much higher density of Bactrian camels and a larger human population, as well as a more active live camel trade and frequent animal transportation as part of the animal husbandry and tourist industries. Therefore, given the highly threatening zoonotic potential of MERS-CoV, we carried out a serological and virological surveillance study in the camel herds of the Alxa, Bayan Nur, and Ordos areas of the IMAR from 26 July to 1 August 2015. We investigated five herds (80 camels sampled) in Alxa, three herds (60 camels sampled) in Bayan Nur, and two herds (50 camels sampled) in Ordos (Table 1). Serum and nasal swab samples were collected from each camel.

The presence or absence of MERS-CoV neutralizing antibodies in the serum samples was determined by using a recombinant chimeric vesicular stomatitis virus (VSV), rVSVΔG/S-eGFP. This virus was generated by using reverse genetics and was based on rVSV-eGFP, a recombinant VSV expressing enhanced green fluorescence protein (eGFP).^8^ In the genome of rVSV-eGFP, the ORF of VSV G was replaced with that of MERS-CoV spike protein (S). rVSVΔG/S-eGFP can infect host cells by utilizing MERS-CoV S to mediate viral attachment and entry; expression of eGFP is an indicator of the infection. Serum from a recombinant Newcastle disease virus expressing MERS-CoV S (rNDV-MERS-CoV-S)-immunized camel served as a control. The method for generating rNDV-MERS-CoV-S was described previously.^9^ Neutralization titers were expressed as the reciprocal of the highest dilution of serum that showed at least 50% inhibition of infection with rVSVΔG/S-eGFP. All serum samples and the control serum from pre-immunized camels had neutralization antibody titers against rVSVΔG/S-eGFP of less than 1:2. In contrast, the control serum from the rNDV-MERS-CoV-S immunized camel yielded neutralization antibody titer of 1:512. An enzyme-linked immunosorbent assay was also carried out to test for MERS-CoV-specific antibodies in serum samples. Vero E6 cells were infected with rVSVΔG/S-eGFP, and the lysate of infected Vero E6 cells was used as the coating antigen for the ELISA. Specific antibody binding to MERS-CoV S was detected with HRP-conjugated Protein A and visualized by adding 3,3′,5,5′-tetramethylbenzidine substrate. All serum samples and control sera from pre-immunized camels yielded OD_450_ values of between 0.17 and 0.29; however, the control serum from the post-immunized camel yielded OD_450_ value of 0.51. The swab samples were tested by using real-time polymerase chain reaction (RT-PCR) targeted to ORF1a of the MERS-CoV genome in accordance with the World Health Organisation protocol.^10^ The results showed that all of the samples were negative for MERS-CoV ORF1a RNA.

In the present study, a total of 190 Bactrian camels from 10 herds were sampled in three areas of the West IMAR of China, where over 40% of the Bactrian camels in China are raised. All 190 serum and swab samples were negative for MERS CoV S-specific neutralizing antibody, ELISA antibody, and MERS-CoV RNA. Our results indicate that there is no MERS-CoV circulating among Bactrian camels in the West IMAR. The absence of MERS-CoV infection in Bactrian camels in other areas of China should be confirmed through further surveillance studies.

## Figures and Tables

**Table 1:** Serological and virological surveillance of Bactrian camels in the West IMAR of China

		Age, years						
Herd NO	Area	≤1	2–4	≥5	Samples /herd NO	VNT (log2)	ELISA (OD_450_)	RT-PCR
1	Alxa	2	4	9	15/20	<1	0.21–0.26	–
2	Alxa	0	3	13	16/28	<1	0.17–0.25	–
3	Alxa	0	4	10	14/45	<1	0.20–0.27	–
4	Alxa	3	6	16	25/65	<1	0.18–0.24	–
5	Alxa	0	0	10	10/18	<1	0.21–0.29	–
6	Bayan Nur	0	5	20	25/40	<1	0.20–0.28	–
7	Bayan Nur	0	3	17	20/36	<1	0.19–0.26	–
8	Bayan Nur	2	2	11	15/32	<1	0.21–0.27	–
9	Ordos	0	4	28	32/66	<1	0.17-0.28	–
10	Ordos	0	1	17	18/33	<1	0.22–0.29	–
Total		7	32	151	190/383			
Pre-immu. sera					1/5	<1	0.17	ND
Post-immu. sera					1/5	9	0.51	ND

ND, not done; pre-immu.sera, camel sera before rNDV-MERS-CoV-S immunization; post-immu.sera, rNDV-MERS-CoV-S-immunized camel sera; VNT, virus neutralization titer; –, negative for MERS-CoV RNA.
